# 

**Published:** 1864-11

**Authors:** 


*Contents.*

ORIGINAL COMMUNICATIONS.......................................... 177
1. The Effects of the Hunterian Method of Ligation on Inflammation, by Daniel F. Wright, Surgeon P. A. C. S. 2. The Climate and Topography of Aiken, South Carolina, in their Relation to Phthisis, by E. S. Gaillard, Surgeon and Medical Inspector. 3. Report of Forty-Eight Cases of Hospital Gangrene and Hospital Erysipelas Treated in General Hospital, Division No. 1, Charlottesville, Va., from June 1st, 1864 to 1st October, 1864, by Acting Assistant Surgeon C. R. Harris, in charge of Gangrene and Erysipelas Ward. 4. Cases of Gunshot Injury Requiring Ligation of the Artery. (From Reports in Surgeon-General's Office.) Collated by H. L. Thomas, M. D. 5. On Treatment of Hospital Gangrene, by Circumscribing Wound with Knife, by T. J. L. De Yampert, Surgeon P. A. C. S. Newsom Hospital, Georgia.
EDITORIAL.......................................................... 187
CHRONICLE OF MEDICAL SCIENCE.................................. 187
On the Absorption of Dead Bone, by Wm. Scovell Savory, Esq., F. R. S., Assistant-Surgeon to St. Bartholomew's Hospital. Royal Medical & Chirurgical Society, April, 1864. On a New Operation for obtaining union of an Ununited Fracture, with Remarks on its Application in Certain Cases of Recent Fracture, by E. R. Bickersteth, F. R. C. S., Surgeon to the Liverpool Royal Infirmary.
INTERESTING ANALYSES OF ARTICLES IN FOREIGN JOURNALS.......................................... 190

